# Components of Impulsivity in Gambling Disorder

**DOI:** 10.1007/s11469-015-9572-z

**Published:** 2015-07-24

**Authors:** David C. Hodgins, Alice Holub

**Affiliations:** Department of Psychology, University of Calgary, 2500 University Drive NW, Calgary, AB T2N 1N4 Canada; Alberta Children’s Hospital, Calgary, AB Canada

**Keywords:** Gambling disorder, Impulsivity, Sensation-seeking, Response impulsivity, Pathological gambling, Personality

## Abstract

This study examined the structure of impulsivity within gambling disorder. A group of 51 men and 53 women with gambling disorder completed self-report and behavioral measures of impulsivity. Principal component analyses found two factors. The first was interpreted as measuring trait impulsivity. This factor correlated with problem gambling severity, presence of comorbid mental health and substance use disorders, history of brain injury, and was higher in Aboriginal participants. The second factor had high loadings on the self-reported sensation-seeking scales and the behavioural measures of response impulsivity. This factor correlated with overall gambling involvement but not with indicators of pathology. Higher scores were associated with younger age. These results are consistent with an evolving model of the etiology of disordered gambling that suggests that sensation-seeking is related to gambling involvement but that trait impulsivity and mental health struggles are associated with the development of gambling disorder.

Impulsivity is a complex multidimensional construct that is associated with numerous mental disorders including personality disorders such as antisocial and borderline disorders, attention deficit, bipolar, eating, substance use and gambling disorders (Congdon and Canli 2005; Sharma et al. 2014). Broadly defined, impulsivity includes the tendency to react rapidly in decision-making or behaviour with a lack of forethought. The construct encompasses personality trait, cognitive, and behavioural components and involves both normal and abnormal individual differences of degree and severity of consequences. Although a variety of more specific conceptualizations of impulsivity have been proposed, there is little consensus on the overall definition, its components or its boundary with related personality traits and cognitive abilities (Sharma et al. 2014). Impulsivity has been called an “artificial umbrella term” given that it lacks a clear and consistent definition across studies (Whiteside and Lynam 2001). Moreover, at the clinical level, impulsivity manifests itself differently in different disorders such that impulsivity associated with eating disorder, for example, is different than the impulsivity associated with attention deficit disorder (Congdon and Canli 2005). Therefore, understanding the structural components of impulsivity within disorders is important in order to understand its implications for treatment and prevention efforts.

Gullo et al. (2014) recently reviewed the broad literature examining the personality trait, cognitive and behavioural aspects of impulsivity in substance use disorders. They argued that there is emerging evidence across a number of diverse research areas indicating two core interconnected processes: an approach impulse process and an inhibitory process. The approach or choice impulse is conceptualized as reward sensitivity and sensation-seeking at the personality trait level, and delay discounting and impulsive choice at the cognitive and behavioral levels. The inhibitory process, or response impulsivity (Potenza and Taylor 2009), is tapped in the popular impulsivity self-report scales (e.g., Barrett Impulsiveness Scale, Eysenck Impulsivity Scale), and at the behavioural level in motor disinhibition and rapid response tendencies.

Gullo et al. (2014) reviewed the evidence for differential predictive validity of measures of these two processes in the substance use disorder area. Overall, results indicated that choice impulse is related to earlier onset of drinking and positive drinking expectancies, whereas response impulsivity is linked to problematic substance use and impairment of control. These processes appear to have neurobiological underpinnings; namely in the mesolimbic and orbital frontal cortex, anterior cingulate cortex, insula, and inferior frontal cortex areas (Aron et al. 2007; Gullo et al. 2014; Gullo and Potenza 2014; Grant and Chamberlain 2014), and generally are thought to be genetically influenced (Bezdjian et al. 2011; Gullo et al. 2014; Ersche et al. 2010).

Similar two factor conceptualizations have been identified by others (Kraplin et al. 2014a; Grant and Chamberlain 2014; Dawe et al. 2004). However, another influential conceptualization of impulsivity resulted from a line of work by Whiteside and Lynam (2001) who described a four factor analytically-derived model that was subsequently extended to five factors (Cyders et al. 2007): negative urgency, positive urgency, sensation seeking, lack of premeditation, and lack of perseverance. The sensation-seeking and lack of premeditation factors are similar to the approach choice and inhibitory response core processes respectively, and Gullo et al. (2014) argued that negative and positive urgency were also both aspects of the inhibitory process. Lack of perseverance, on the other hand, appears to be a separate construct not identified in the two factor model. Gullo maintained that lack of perseverance is not strongly linked to substance abuse (Gullo et al. 2014), although the supporting evidence is limited.

This lack of consensus on the components of impulsivity extends into the gambling area. Grant and Chamberlain (2014) recently compared research assessing cognitive and behavioural elements of impulsivity in substance use and behavioural addictions, including gambling disorder. The review used a two factor conceptualization similar to Gullo et al. (2014), and concluded that there is considerable overlap between substance use and behavioural addictions in their cognitive and neural substrates. Although there are some inconsistent findings, both choice and response inhibition were found to be deficient in gambling disorder in cross-sectional data. However, there is a lack of prospective studies identifying whether these deficiencies predate gambling disorder (Grant and Chamberlain 2014).

The association between gambling and trait impulsivity has been studied more extensively than the relationship with the cognitive and behavioural components. A large group of studies have demonstrated a positive cross-sectional association with self-report measures of impulsivity and gambling involvement and gambling disorder in both clinical (Grall-Bronnec et al. 2012; Ledgerwood and Petry 2010; Billieux et al. 2012; Albein-Urios et al. 2012; Whiteside et al. 2005; Hwang et al. 2012; Michalczuk et al. 2011) and nonclinical populations (Cyders and Smith 2008; Estevez et al. 2013; Marmurek et al. 2013). Only a minority of studies fail to find a significant relationship (e.g., Petry 2001; Langewisch and Frisch 1998; Lejoyeux et al. 1998). In addition to these cross-sectional data, there is also some preliminary evidence that impulsivity indicators in childhood are prospectively associated with gambling disorder in emerging adults (Slutske et al. 2005; Dussault et al. 2011; Auger et al. 2010).

In order to advance the field, a more nuanced understanding of the structure of impulsivity in gambling disorder is important (Sharma et al. 2014). To date, only two studies have attempted to explore the different components (i.e., factor structure) of impulsivity, specifically, in gambling disorder. Kraplin and colleagues (Kraplin et al. 2014a) conducted exploratory factor analyses in a mixed group of treatment-seeking individuals (disordered gambling, alcohol use disorder, Tourette syndrome) and healthy controls. Using a battery of mostly behavioural and neuropsychological tasks assessing impulsivity and executive functioning, they found four factors, all of which were elevated in the substance use and gambling disorder subgroups versus the healthy controls. The factors were: self-reported impulsivity, choice impulsivity, motor impulsivity and prepotent response impulsivity. Factor scores did not differ between the alcohol and gambling subgroups and only the choice impulsivity score distinguished the gambling and Tourette subgroups (gamblers were more elevated). The authors acknowledged that it was unfortunate that the subgroups were too small to examine factor invariance across the disorders.

Another examination of the structure of self-reported impulsivity used a sample of college students with a range of involvement in gambling (Ginley et al. 2014). Factor analyses revealed three factors: behavioural activation (drive, fun-seeking), which unexpectedly correlated negatively with gambling frequency; preference for stimulation, which correlated positively with gambling frequency; and inhibition control, which had a very weak but statistically significant relationship with gambling problem severity. The authors acknowledged that the low base rate of gambling and problem gambling in the sample likely affected the validity and reliability of the results.

The present study explores the factor structure of the impulsivity construct within a group of non-treatment-seeking individuals diagnosed with gambling disorder (American Psychiatric Association 2013). A variety of personality, cognitive, and behavioral measures of impulsivity representative of both choice approach and response inhibition impulsive processes were administered, along with a variety of external measures, to help explicate the meaning of the identified dimensions. Because existing research on the structure of impulsivity in pathological gambling is lacking, an exploratory versus hypotheses-testing approach was adopted.

## Methods

### Participants and Procedure

Community-dwelling adults (*N* = 104) were recruited through media advertisement. Inclusion criteria were age 18 or older, and a score of 8 or greater on the Problem Gambling Severity Index (Ferris and Wynne 2001), indicating problem gambling. Written informed consent was obtained during the laboratory session. The session length was 90 to 120 min (M = 105), and consisted of a variety of interview, self-completion, and cognitive tasks. One task, the Iowa Gambling Task (Bechara et al. 2000), involved simulated gambling and, for ethical reasons, participants were given the choice of not completing it if they were attempting to abstain from gambling. Because of a significant amount of missing data, that measure is excluded from these analyses.

### Measures

Participants provided demographic information, a gambling history, and a neurological medical history via interview. The gambling history included frequency of involvement in 15 different types of gambling over the past six months as well as identification of the most problematic types. Stinchfield’s measure of pathological gambling (Stinchfield 2003) provided a past year DSM symptom count which was used to make a diagnosis of DSM-5 gambling disorder. Problem gambling severity was also measured using the Problem Gambling Severity Index (PGSI), a nine-item scale assessing severity of problems in the past year. The PGSI is widely used with good reliability and validity and cut-off of 8 or greater indicates problem gambling (Currie et al. 2012).

A number of self-report scales were administered. The *Barrett Impulsiveness Scale (BIS)*, version 11 (Patton et al. 1995) is a 30-item measure that provides three subscales: cognitive impulsiveness (*I have racing thoughts*), motor impulsiveness (*I act on impulse*), and non-planning (*I do things without thinking*). Internal reliability in this sample was α = .62, .68, and .72.

The *Eysenck Impulsivity Scale* (I_7_) (Eysenck and Eysenck 1978) has 54 items and three subscales. Impulsiveness (unplanned behaviour; e.g., *Before I start a complicated job or project, I tend to make careful plans*), venturesomeness (sensation-seeking; e.g., *I will try anything once*) and empathy (*Do you often get emotionally involved with your friends problems*?). The first two subscales are measures of aspects of impulsivity and the third subscale, which provides “buffer” items, was excluded from these analyses. Internal reliability in this sample was α = .86 for venturesomeness and .86 for impulsiveness.

The *Impulsive Sensation Seeking Scale* of the Zuckerman-Kuhlman Personality Questionnaire III (I_ss_, Zuckerman et al. 1993) has 19 items, 8 measuring impulsivity (inability to plan, failure to deliberate, *I very seldom spend much time on the details of planning ahead*) and 11 measuring sensation-seeking (preference for exciting, novel, and unpredictable situations; *I like doing things for the thrill of it*). Internal reliability in this sample was α = .80 for impulsivity and .77 for sensation-seeking.

The *Adult ADHD Rating Scales- Short Version* (Conners et al. 1999) is a 26-item self-report scale assessing hyperactivity/restlessness, inattention/memory problems, impulsivity/emotional liability, and problems with self-concept. The first three subscales were included as measures of aspects of impulsivity and the self-concept scale was used as an external validity scale in this study. Research has confirmed the four factor structure of this scale as well as good concurrent validity compared with observer ratings of ADHD symptoms. Internal reliability in this sample was hyperactivity/restlessness, α = .79, inattention/memory problems, α = .74, impulsivity/emotional liability, α = .75, and problems with self-concept α = .90.

Conner’s *Continuous Performance Test* (CPT; Conners 1995) was used to assess cognitive impulsivity. The CPT is administered via computer and requires the participant to push a key on the keyboard only in response to a designated target. Two measures of performance were included in this study: number of commission errors, indicting impulsive responding to expected stimuli and reaction time, with low scores (quick response) indicating lack of sustained attention and vigilance. The CPT has been found to be a stronger indicator of sustained attention than other stop signal tasks (Epstein et al. 2001).

Measures of other constructs included for external validation included the *Tridimensional Personality Questionnaire* (TPQ; Cloninger et al. 1991), a 98-item self-report scale that assesses the three personality dimensions described in Cloninger’s model of personality (Cloninger et al. 1993): novelty-seeking, harm avoidance, and reward dependence. The scale does not assess impulsivity directly, but Cloninger has suggested that impulsivity is represented by high levels of novelty-seeking and low levels of harm avoidance. For purposes of this study, an impulsivity index was created by subtracting the harm avoidance score from the novelty-seeking score.

The *Wisconsin Card Sorting Test* (WCST; Heaton 1993) was administered via computer. The WCST, a measure of executive functioning, requires that the participant sort cards into piles using card features based upon minimal and shifting information. The percentage of preservative errors, indicting lower ability to shift to new rules, was used in this study. Selected modules of the Structured Clinical Interview for DSM-IV Diagnosis (SCID; First et al. 1997) provided substance use and impulse control disorders and ADHD diagnoses.

### Analytic Approach

Exploratory principal components analysis (PCA) was conducted using SPSS, V 21. The variables included were: I7 impulsiveness, I7 venturesomeness, CPT Commissions, CPT reaction time, I_ss_ sensation-seeking, I_ss_ impulsiveness, BIS motor, BIS attention, BIS non-planning, ADHD hyperactivity/restlessness, ADHD inattention/memory problems, and ADHD impulsivity/emotional liability. Variables were examined for outliers and multicollinearity, and no violations were uncovered. Kaiser-Meyer-Olkin Measure of Sampling Adequacy and Bartlett’s Test of Sphericity were used as indicators of the factorability of the set of variables in this sample. The number of factors rotated was based upon the distribution of Eigenvalues (scree test) and parallel analysis (Horn 1965; O’Connor 2000). Oblim and Varimax rotations were performed and because the factors were not significantly correlated, Varimax rotated loadings are reported. The factor scores for the final solution were correlated with external validation variables using Pearson correlations for continuous and t-tests for dichotomous variables.

## Results

Of the 104 participants, 51 % were female. The mean age was 43.5 years (SD = 13.2, range 19 to 75) and the mean years of education was 14.6 (SD = 5, range 5 to 20). In terms of marital status, 28.8 % were single, 38.5 % married or common-law, 28.8 % divorced and 2.9 % widowed. Over half (56.7 %) worked full or part-time, 12.5 % were unemployed and 11.5 % retired. In terms of ethnicity, 72.0 % reported Caucasian, 20.2 % Aboriginal, 5.8 % Asian, and 3.0 % mixed.

All participants exceeded the cut-off for problem gambling of 8 on the PGSI (M = 14.4, SD = 4.8), and met the DSM-5 criteria for gambling disorder (M = 7.1 symptoms, SD = 1.8, range 4 to 8). Participants reported engaging in a mean of 6.5 types of gambling (SD = 3.6). About a third (35 %) reported problems with video lottery terminals, 23 % slot machines, 13 % casino games, and 6 % bingo.

Unexpectedly, 53 % of participants reported a history of brain injury during the clinical interview, with 19 % reporting two or more injuries, 36 % reporting experiencing a loss of consciousness, and 31 % reporting being hospitalized as a result of the injury. Regarding cardiovascular problems, 8.7 % reported a history of stroke or heart attack, and participants also reported a range of other problems including most frequently migraines and headaches (11.5 %), mood disorders (5.8 %), angina or hypertension (5.8 %), and diabetes (4.8 %).

Table 1 provides the mean (SD) and Pearson correlations among the variables included in the PCA. The Kaiser-Meyer-Olkin Measure of Sampling Adequacy was .80 and Bartlett’s Test of Sphericity produced a *χ*^2^ (66) = 592.6, *p* < .0001, indicating good factorability of these variables. The PCA yielded three Eigenvalues greater than one, accounting for 67.5 % of the variance. Examination of the distribution of the Eigenvalues and the parallel analyses test indicated that two factors be rotated. Varimax rotation yielded a clear factor loading pattern. Table 2 displays the rotated factor loadings and communalities. The first rotated factor, accounting of 37.1 % of the variance, had high loadings from the scales measuring self-reported impulsivity and ADHD symptoms and was interpreted as measuring trait impulsivity. The second factor, accounting for 19.2 % of the variance, had high loadings from the self-reported sensation-seeking scales and the two CPT behavioural measures of response impulsivity.

**Table 1:** Descriptive statistics and correlation matrix

Measure	M	SD	1	2	3	4	5	6	7	8	9	10	11	12
1. I7 - Impulsiveness	10.50	4.80		.13	−.02	.25	.76	.39	.60	.53	.69	.42	.51	.47
2. I7 - Venturesomeness	8.60	4.50			−.21	.17	.28	.72	.13	.04	.00	−.02	.15	.02
3. CPT-RT	47.50	15.00				−.50	−.04	−.31	.03	−.07	.16	−.03	−.17	−.08
4. CPT-Commissions	52.00	12.00					.23	.37	.18	.34	.12	.33	.23	.18
5. Iss Imp	.50	.30						.41	.63	.48	.68	.39	.40	.35
6. Iss Sen	.60	.30							.24	.26	.16	.12	.38	.24
7. BIS Motor	26.70	4.10								.47	.63	.32	.31	.35
8. BIS Attention	18.30	3.20									.43	.45	.65	.43
9. BIS None planning	28.70	4.70										.39	.29	.37
10. ADHD-Inattention/Memory	5.20	2.80											.41	.57
11. ADHD-Hyperactivity/Restlessness	7.10	3.30												.54
12. ADHD-Impulsivity/Emotional lability	4.50	2.70												

Correlations > .15 are significant at *p* < .05

*BIS* Barratt impulsivity scale, *CPT* Conner’s continuous performance test, *I7* Eysenck impulsiveness scale, *Iss Imp* sensation seeking scale - impulsiveness, *Iss Sen* Sensation seeking scale - sensation seeking, *RT* Reaction time

**Table 2:** Rotated component matrix of impulsivity measures

Measure	Component	
	Trait Impulsivity	Sensation-seeking/Response impulsivity
I7 - Impulsiveness	**0.832**	0.156
BIS None planning	**0.796**	- 0.120
Iss Imp	**0.764**	0.236
BIS Attention	**0.739**	0.145
BIS Motor	**0.718**	0.092
ADHD-Inattention/Memory	**0.676**	0.010
ADHD-Impulsivity/Emotional lability	**0.674**	0.071
ADHD-Hyperactivity/Restlessness	**0.648**	0.277
Iss Sen	0.270	**0.821**
I7 - Venturesomeness	0.008	**0.762**
CPT-RT	0.078	**- 0.685**
CPT - Commissions	0.263	**0.589**

*n* = 104

*BIS* Barratt impulsivity scale, *CPT* Conner’s continuous performance test, *I7* Eysenck impulsiveness scale, *Iss Imp* sensation seeking scale - impulsiveness, *RT* Iss Reaction Time, *Sen* Sensation seeking scale - sensation seeking

Table 3 reports the correlations between these rotated factor scores and demographics, and gambling, comorbidity and personality variables. Neither factor was significantly correlated with gender, but sensation-seeking/response impulsivity was negatively correlated with age (younger participants were higher), and Aboriginal participants were more trait impulsive. Overall, the external variables tended to correlate significantly with one but not both of the factor scores.

**Table 3:** Correlations between demographic and clinical characteristics and factors

Variable/Measure	Component	
	Trait impulsivity	Sensation-seeking/response impulsivity
Demographics		
Aboriginal	0.22*	- 0.03
Age	- 0.13	- 0.44***
Sex (1 = female, 2 = male)	0.15	- 0.13
Comorbidity		
History of brain injury	0.26**	0.06
Loss of consciousness	0.27**	0.10
Brain Injury - Hospitalization	0.28**	0.19
History of stroke/Heart attack	- 0.04	0.07
ADHD	0.30**	0.03
Alcohol use disorder	0.18	0.10
Drug use disorder	0.31***	0.12
Impulse control disorder	0.20*	0.16
Problems with self-concept	0.53***	- 0.10
Gambling		
Number of types of gambling	0.19	0.33***
Frequency of casino games	0.10	0.38***
PGSI	0.23*	- 0.07
DSM -IV Criteria nPG)	0.32**	0.03
Personality		
WCST-Perseveration	0.14	0.10
TPQ-Harm avoidance	0.36***	- 0.45***
TPQ-Novelty-seeking	0.54***	0.35***
TPQ-Reward dependence	0.08	0.02
TPQ-Impulsivity	0.06	0.53***

*p < .05. **p < .01. ***p < .001.

*PG* Pathological gambling, *PGSI* Problem gambling severity index, *TPG* Tridimensional personality questionnaire, *WCST* Wisconsin card sort test

## Discussion

This study is the first to explore the structure of the broad construct of impulsivity in individuals diagnosed with disordered gambling. Two factors were uncovered, one measuring general trait impulsivity and the other measuring sensation-seeking and CPT performance. The general impulsivity factor was characterized by loadings on the self-report measures designed to assess the overall trait impulsiveness (I7 impulsiveness, Zukerman impulsivity) as well as the three BIS Impulsivity subscales and the three Connors’ ADHD subscales, all of which loaded equally and strongly on this factor. The second factor was characterized by loadings on the two self-report scales measuring sensation-seeking and the two measures derived from the CPT. The CPT commission errors score, indicating impulsive responding to expected visual stimuli, loaded positively, and CPT Reaction Time score, with low scores indicting a lack of sustained attention and vigilance, loaded negatively. This two factor model differs somewhat from previous two factor conceptualizations in the substance abuse literature that group trait impulsivity self-report scales and motor response inhibition indices, such as the CPT measures, together (Gullo et al. 2014). These results group these latter indices with sensation-seeking measures.

The trait impulsivity factor also correlated with problem gambling severity, which is consistent with the large body of literature that has assessed the correlation of many of these scales individually with gambling severity. The sensation-seeking/response impulsivity factor, in contrast, was unrelated to problem gambling severity. Instead, it correlated significantly with number of different gambling activities played in the past six months. These results suggest that one aspect of impulsivity seems to drive involvement with gambling whereas another predicts problematic involvement. This finding is consistent with an evolving model of the etiology of gambling disorder that finds different predictors for high gambling involvement and for addiction (Hodgins et al. 2012). In that analysis, excitement-seeking personality, which is similar to sensation-seeking, was one of the factors associated with higher frequency gambling, but not problematic gambling. That study also found an impulsivity measure, similar to the response impulsivity factor in this study, was associated with gambling disorder. Raylu and Oei (2002) also proposed that impulsivity and sensation-seeking impact different phases of gambling disorders and similar proposals have been made for substance abuse (Ersche et al. 2010). That is, sensation-seeking may stimulate individuals to initiate gambling or substance use. The element of risk, unpredictability, and excitement would make gambling an appealing activity for sensation-seeking individuals. Impulsivity was proposed as a factor that maintains gambling or substance use in the face of losses and other negative consequences.

Neither aspects of impulsivity identified in the PCA correlated with gender but younger people had higher levels of sensation-seeking/response impulsivity. This latter result is also consistent with previous research that found that younger people had greater gambling involvement, but not necessarily problems (Hodgins et al. 2012). Aboriginal individuals had higher trait impulsivity than non-Aboriginals. It is unclear whether Aboriginals in community samples have higher response impulsivity than other groups, but, if so, this may be a vulnerability factor in higher rates of addiction, including gambling (Wardman et al. 2001).

In the current study, the trait impulsivity factor also correlated with almost all of the comorbidity variables, including drug use disorder, ADHD, impulse control disorders, and problems with self-concept. The correlation with alcohol use disorders was positive but not significant. Again, none of these variables was associated with the sensation-seeking/response impulsivity factor. These results are also consistent with the etiological model identified in Hodgins et al. (2012), in which mental health indicators, substance abuse, antisocial personality, and childhood trauma predicted gambling disorder among high frequency gamblers.

A history of brain injury was surprisingly frequent in this sample, given that they were recruited from the community and not selected on this basis. Recent community data from another Canadian province found a prevalence rate of 17 % using similar criteria, although prevalence was associated with drug use and psychological distress, which suggests that prevalence would be significantly higher in these subpopulations (Ilie et al. 2014). Based upon clinical interviews, over half of participants reported a history of brain injury with about one-third reporting loss of conscientiousness and hospitalization. We were unable to corroborate these interview reports with medical records. However, all three of these indicators were significantly correlated with the trait impulsivity factor, but not the sensation-seeking/response impulsivity factor. Direction of causality is unclear – impulsivity may be a sequelae of brain injury but, in contrast, impulsive individuals may be more likely to suffer brain injury due to their impulsive behaviours.. History of stroke or heart attack did not correlate with either factor.

Neither aspect of impulsivity was associated with executive functioning as measured by the WCST. This supports the notion that executive functioning is a separate construct from impulsivity. Previous research on the role of executive functioning in gambling disorder is inconsistent generally (Ledgerwood et al. 2012; van Holst et al. 2010) and specifically in studies using the WCST as a measure (Cavedini et al. 2002; Odlaug et al. 2011). In this instance, individuals performed slightly lower than the normative sample (35 to 45th percentile) but none of the WCST indices correlated with problem gambling severity.

The final set of external correlates examined was the Tridimensional Personality Questionnaire subscales. Although reward dependence is generally thought to be linked to addictive behaviours, it did not correlate with either impulsivity factor in this study. Previous research has also not found that this factor was elevated in disordered gambling, unlike novelty-seeking and harm avoidance (Tavares and Gentil 2007; Skitch and Hodgins 2004; Kim and Grant 2001; Janiri et al. 2007). In the present sample, novelty-seeking correlated positively with both factors. This construct involves the tendency to actively seek excitement and avoid punishment and frustration. Harm Avoidance, which refers to behavioral inhibition and the tendency to develop fears and anxiety, correlated positively with trait impulsivity and negatively with sensation-seeking/response impulsivity.

The strongest correlation was between the unvalidated TPQ impulsivity score that we derived for this study and the sensation-seeking/response impulsivity factor. The TPQ index was calculated by subtracting the harm avoidance score from the novelty-seeking score, based upon Cloninger’s proposal that impulsivity was related to a combination of high levels of novelty-seeking and low levels of harm avoidance. These results suggest that the Cloninger concept of impulsivity is related to sensation-seeking/response impulsivity versus trait impulsivity.

This study represents the first examination of the factor structure of the construct of impulsivity in disordered gambling in a well characterized sample. The sample size was small, but adequate for the analyses conducted (Tabachnick and Fidell 2007). A variety of different types of impulsivity measures were administered, although not all aspects of impulsivity were well represented in this study. A notable omission was any delay discounting task, which is typically shown to be impaired in addiction populations, including gambling disorder (Kraplin et al. 2014b; Petry and Casarella 1999; Shead and Hodgins 2009). It is also unfortunate that few participants agreed to complete the Iowa Gambling Task, which is also often used to assess impaired decision-making in disordered gambling (Brevers et al. 2013). The battery in the current study had a number of behavioral tasks, but it was dominated by self-completion measures.

Another limitation of the design was the lack of a healthy control group. Although available norms for a number of instruments suggested that generally scores were below normal, a control group would be helpful to judge the relative levels of functioning in this group. Assessment of factor invariance between healthy individuals and individuals with gambling disorder would also be informative.

In conclusion, the results of this study suggest that impulsivity in gambling disorder is represented by two independent components. The trait factor uncovered in this study is related to pathological functioning, including problem gambling severity, other comorbid mental health issues, history of brain injury, and high harm avoidance. Focusing on identifying individuals higher in this tendency and providing remediation may be a fruitful focus of prevention and treatment. The other factor, which is associated with low harm avoidance, sensation-seeking, and participation in a wider range of gambling activities, is not associated with pathological functioning.

Many descriptive studies on gambling disorder include a single self-report impulsivity measure. In some assessment domains, multiple scales with the same label have items that focus on different aspects of a construct, which leads to conceptual confusion (e.g., self-control, cognitive distortions). In contrast, these results suggest that any of the common self-report measures is a reasonable proxy for the impulsivity factor associated with pathology. This is particularly good news as longitudinal studies of the onset of gambling disorder most often include these relatively brief indicators of the construct. These results suggest that such measures represent an important aspect of impulsivity. Similarly, measures of sensation-seeking are proxies for the sensation-seeking/response impulsivity factor, and measuring this aspect of impulsivity has incremental validity. Future research is required to cross validate these factors, including additional measures that represent cognitive, personality and behavioral aspects in a more balanced fashion, and in larger samples.
